# Clinical Analysis of Renal Failure Caused by Malakoplakia: A Case Report and Literature Review

**DOI:** 10.3389/fmed.2022.770731

**Published:** 2022-03-03

**Authors:** Zhenting Wang, Jiannan Ren

**Affiliations:** ^1^Department of Urology Surgery, Haikou People's Hospital/Affiliated Haikou Hospital of Xiangya Medical College, Central South University, Haikou, China; ^2^Department of Urology Surgery, The Second Xiangya Hospital, Central South University, Changsha, China

**Keywords:** malakoplakia, bladder malakoplakia, chronic renal insufficiency, pathological examination, nephrectomy

## Abstract

**Background:**

A 52-year-old middle-aged woman developed fatigue, poor appetite and elevated CRE levels 6 months ago without known causes of these symptoms. After repeated anti-infective and renal function improvement treatment, her symptoms did not significantly improve and local progress. Pathological examination confirmed soft spot disease of left kidney and bladder.

**Tests:**

Plain computed tomography (CT) scan of the lungs and urinary system; CT enhancement of the abdomen and pelvis, magnetic resonance (MR) enhancement of the abdomen and pelvis; positron emission tomography (PET)-CT; ultrasonography of the urinary system; cystoscopy, biopsy; and laboratory examination including urine routine and culture, urine protein quantification, cytology and culture of drainage fluid smear, routine blood test, and assessment of C-reactive protein, sedimentation, procalcitonin, liver function, renal function, electrolytes, thyroid function, parathyroid function, and cardiac enzymes.

**Diagnosis:**

Left-sided renal and bladder malakoplakia, chronic renal insufficiency (CKD stage 4), renal anemia, complicated urinary tract infection (*Escherichia coli* + smooth bacilli), chronic urinary retention, abscess of the left psoas major and iliac fossa, type II diabetes mellitus, grade III hypertension (very high risk), and post cholecystectomy.

**Treatment:**

Hospitalized urinary catheterization, anti-infective treatment, diagnostic anti-tuberculosis treatment, gastrointestinal dialysis, correction of anemia treatment, nutritional support, left lumbar enlargement, iliac fossa abscess puncture drainage, left nephrectomy.

## Medical History Reporting

A 52-year-old middle-aged female patient had fatigue and poor appetite along with progressive elevation in the CRE level 6 months ago and complained of occasional cough; no fever, nausea, or vomiting; low back pain and discomfort; and frequent, urgent, and painful urination, with a weight loss of 12.5 kg from symptom onset. Laboratory tests showed an HGB level of 48 g/L and a CRE level of 157 μmol/L, and bone marrow aspiration indicated active myelodysplasia. The patient's symptoms did not markedly improve after receiving several treatments for protecting renal function and correcting anemia. During patient interview, it was found that she had recurrent urinary retention for the past 3 years and had received an indwelling catheter several times.

The patient had a history of hypertension for more than 10 years with a maximum blood pressure of 185/95 mmHg, which was under control (in the normal range) without medication at the time of the study. She was diagnosed with type II diabetes mellitus 3 years ago and did not receive systemic treatment, during which time her blood glucose level was not monitored regularly. More than 10 years ago, the patient underwent cholecystectomy for gallbladder stones. She denied having a history of infectious diseases such as viral hepatitis and HIV infection, history of tuberculosis and close contact with tuberculosis patients, and travel to areas with a high prevalence of epidemics and infectious diseases. There was no history of drug or food allergy, smoking, and alcohol consumption.

**Figure 1:**
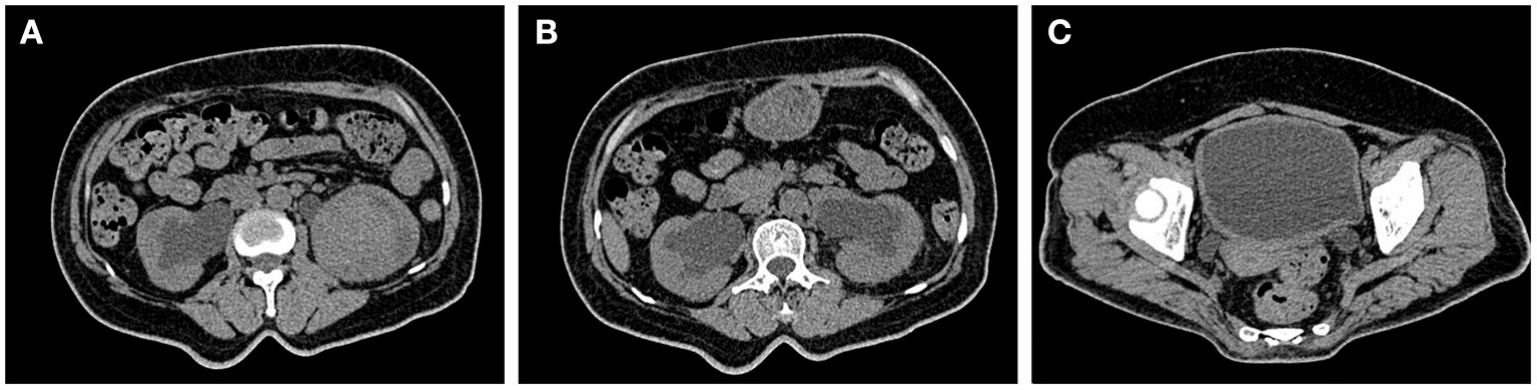
Space occupying lesions of the left kidney. **(A)** Inflammatory mass (such as combination) or tumor may be considered. **(B,C)** Homogeneous thickening of the bladder wall with bilateral dilatation of the ureter and bilateral hydronephrosis.

Before admission, the patient's renal function showed progressive decline. Two days before admission, her renal function was reviewed: CRE level, 404 μmol/L; routine blood tests: white blood cell count, 16.6^*^10^9^/L; N level, 90.5%; HBG level, 68 g/L; random blood glucose level, 13.4 mmol/L. The results of her laboratory tests such as liver function, thyroid function, and tumor markers; chest radiography; and electrocardiography were not markedly abnormal. She was admitted to the Department of Nephrology of our hospital. Upon physical examination, the following findings were observed: body temperature, 36.3°C; pulse, 96 beats/min; respiration, 20 breaths/min; blood pressure, 118/73 mmHg. Furthermore, malnutrition, anemic appearance, no pressure pain and rebound pain in the entire abdomen, no percussion pain in both kidney areas, negative neurological examination findings, and no positive signs in the rest of the general physical examination were noted. After admission, whole-blood cytology, urine sedimentation, and other laboratory tests were repeated (Table 1): Renal function tests indicated that the CRE level was higher than that before; liver function tests indicated that the albumin level was 28.2 g/L; urine sedimentation indicated the following findings: urine protein qualitative (+), urine leukocyte esterase (+++), urine occult blood (+++), and 24 h total urine protein level of 1705.59 mg/d; urine culture showed the presence of *Escherichia coli* and smooth bacilli; and coagulation function, hepatitis complete set, HIV+TP, complement, lupus complete set, vasculitis 6, anti-ENA antibodies, blood and urine coagulation electrophoresis, tumor markers, and thyroid function showed no significant abnormalities. Abdominal ultrasonography suggested diffuse bilateral renal parenchymal lesions, a very hypoechoic mass under the left kidney considered as an occupying lesion, bilateral dilated ureters throughout, and moderate hydronephrosis in both kidneys. The bladder wall was thickened and appeared gross. Cardiac ultrasonography suggested tricuspid regurgitation (mild). CT scan of the urinary system suggested a left renal-occupying lesion, considering either an inflammatory-type mass (if combined) or a tumor, and homogeneous thickening of the bladder wall with bilateral full ureteral and bilateral hydronephrosis dilatation (Figure 1). The patient was considered to have a diagnosis of chronic renal insufficiency (possible obstructive nephropathy and complicated urinary tract infection) and was treated with indwelling catheterization, anti-infection treatment, enteral dialysis, renal function-improving treatment, and anemia-correcting treatment.

**Table 1:** Laboratory biochemical test results.

**Test items (unit)**	**Detection value**			**Reference value**
	**First detection**	**Preoperative detection**	**Postoperative detection**	
Leukocyte count (10^9^/L)	12.2	13.86	7.63	3.50–9.50
Absolute value of neutrophils (10^9^/L)	10.32	13.01	5.46	1.80–6.30
Neutrophil ratio (%)	84.6	93.9	71.5	40.00–75.00
Hemoglobin (g/L)	78	86	116	115–150
Hematocrit (%)	26.4	28	34.5	35.00–45.00
Creatinine (umol/L)	462.4	154.5	238.8	44.0–133.0
Sodium (mmol/L)	132.3	139	139.8	137.0–147.0
Potassium (mmol/L)	4.79	4.02	4.26	3.50–5.50
Calcium (mmol/L)	2.35	2.16	2.5	2.11–2.52

**Figure 2:**
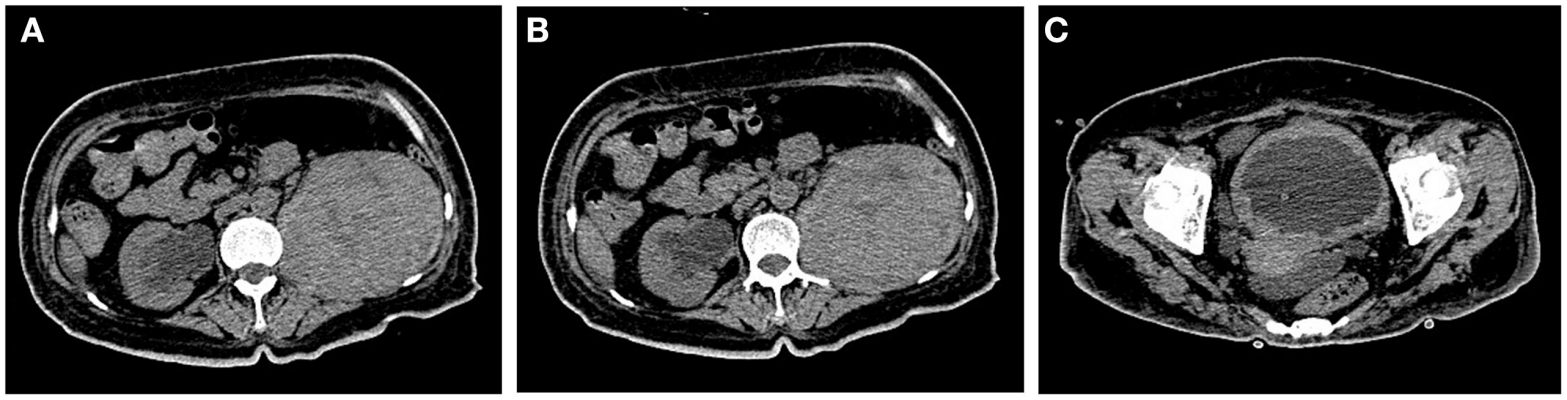
**(A)** The left renal mass was larger than before and divided from the left psoas major. **(B)** The right renal pelvis and ureteral effusion were less dilated than before. **(C)** The bladder wall was uniformly thickened more markedly than before.

During the treatment period, the patient developed unexplained persistent pain in the left sacroiliac joint with limited movement of the left lower limb, which gradually worsened, and had difficulty in walking for a short time. Re-examination indicated that the levels of inflammatory indexes were higher than those before (Table 1), and the tuberculosis spot test, PPD skin test, and antacid staining all yielded negative findings. The G test and GM test also showed no abnormalities. Repeated urological ultrasonography showed an occupying lesion in the lower left kidney and mild hydronephrosis in both kidneys. A plain CT scan of the pelvis revealed swelling of the left psoas major, thickening and dilatation of the left lower and middle regions of the ureter, and thickening of the bladder wall of a nature to be determined (Figure 2). After a hospital-wide consultation, antibiotics were changed to a combination of piperacillin/tazobactam, moxifloxacin, and voriconazole as an anti-infection treatment, and renal MR enhancement and PET-CT were completed according to the consultation. The renal MR enhancement suggested the possibility of left renal tuberculosis with abscess of the left psoas major, accompanied by the surrounding fatty interstitial infiltration, multiple lumpy abnormal signals in the right kidney, considering retrograde infection, possible tuberculosis cold abscess, and possible bilateral ureteritis. PET-CT showed a probable left renal abscess involving the adjacent left psoas major.

**Figure 3:**
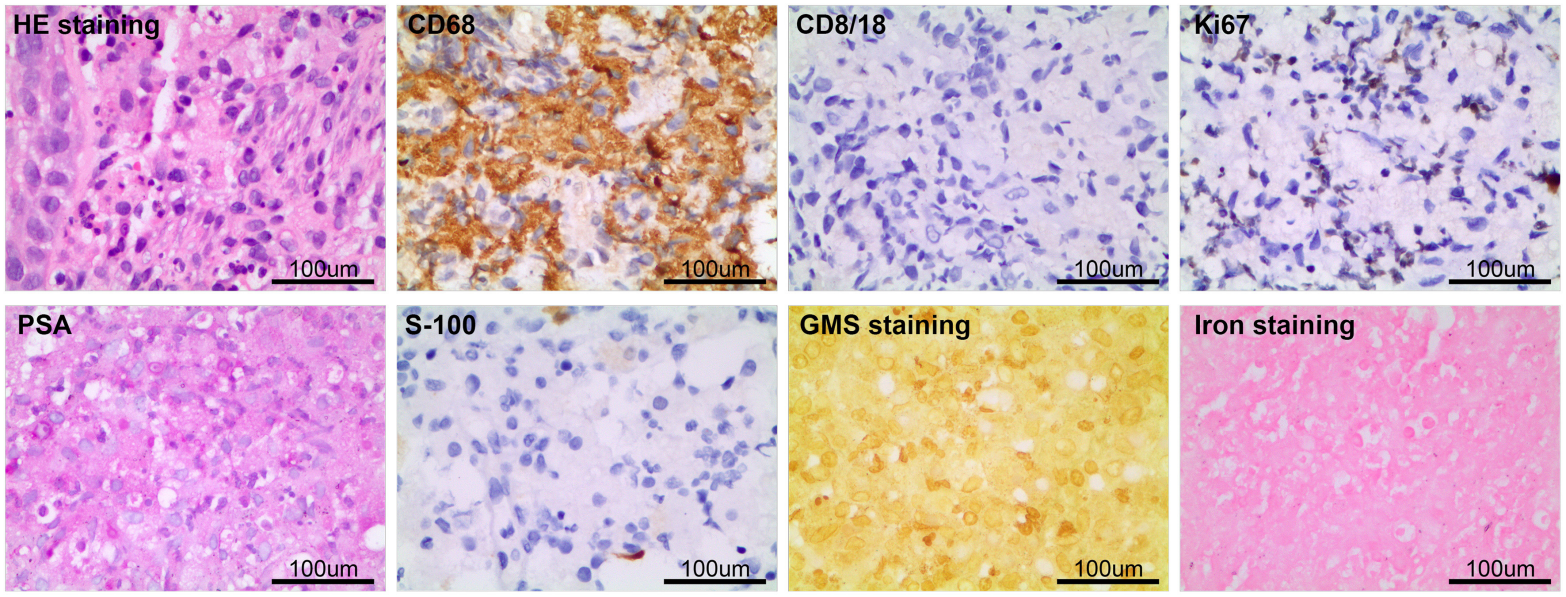
The pathological examination of left kidney puncture showed proliferative fibrous tissue with a small amount of lymphocyte and plasma cell infiltration, PAS and hexamine silver staining (–). Histopathological examination of bladder mass showed that there were many eosinophilic granular tissue cells in the lamina propria of mucosa, scattered in small circular basophilic inclusions, consistent with bladder soft spot disease. Immunohistochemistry: CK (epithelium +), CD68 (+), CD8/18 (epithelium +), GATA (epithelium +), S100 (scattered +), Ki-67 (5%); Special staining: pas (+).

After considering the patient's condition and obtaining the consent of the patient and her family, the patient was treated with isoniazid combined with rifampicin as anti-tuberculosis treatment, and CT-guided puncture and drainage of abscess of the left psoas major and left iliac fossa as well as left renal mass biopsy were performed. Cystoscopy suggested diffuse bulging in the bladder, and biopsy of the bladder mass was then performed. The puncture drainage fluid was cultured for *E. coli*, and the results of the tests related to *Mycobacterium tuberculosis* were negative. Puncture biopsy of the left renal mass suggested proliferating fibrous tissue with mild infiltration of lymphocytes and plasma cells, no granulomatous nodules, and no evidence of malignancy. Special stains [PAS and hexamine silver staining (–)] showed no evidence of fungi and *M. tuberculosis*. Histopathological examination of the bladder mass biopsy indicated many microscopic histiocytes with eosinophilic granular cytoplasm in the lamina propria of the mucosa, and scattered small round basophilic inclusion bodies were seen, which is consistent with bladder malakoplakia. Immunohistochemistry indicated the following findings: CK (epithelial +), CD68 (+), CK8/18 (epithelial +), GATA (epithelial +), S100 (scattered +), Ki-67 (5%). Special stains indicated PAS (+) (Figure 3).

**Figure 4:**
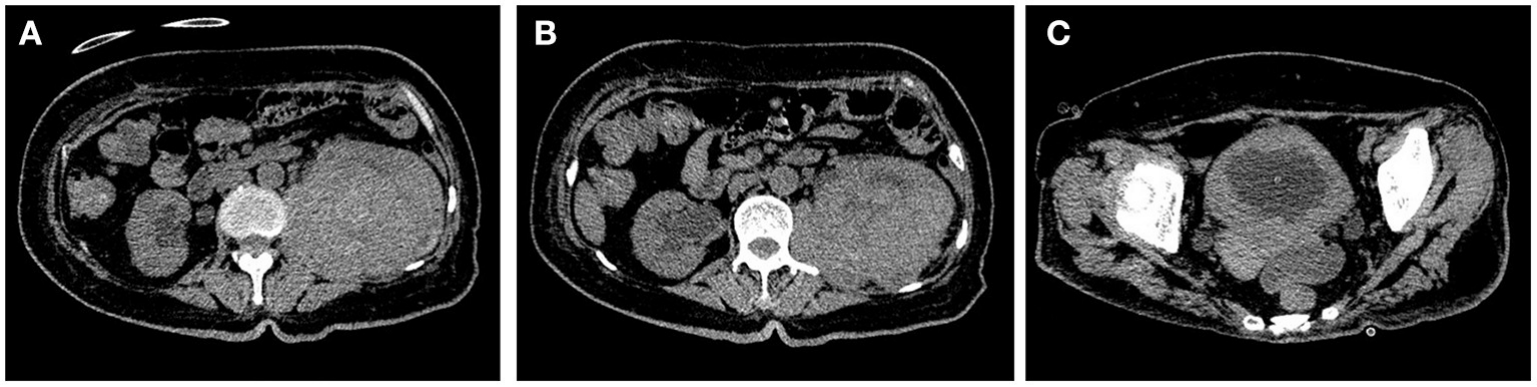
**(A,B)** Bilateral ureteral and bilateral hydronephrosis decreased gradually, and the left renal mass increased continuously. **(C)** The bladder wall was continuously thickened, and the bladder volume was further reduced.

**Figure 5:**
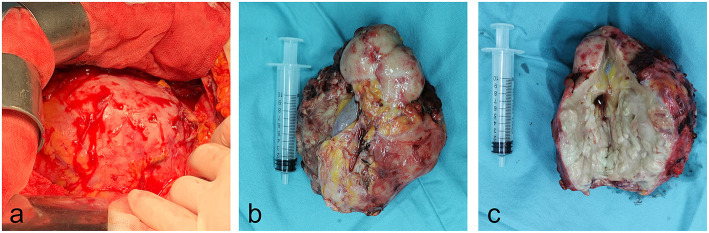
Gross photos of specimens during and after operation. **(a)** The left kidney swelling increased significantly during the surgery. **(b,c)** The overall appearance and tissue section of the left kidney specimens cut during operation.

**Figure 6:**
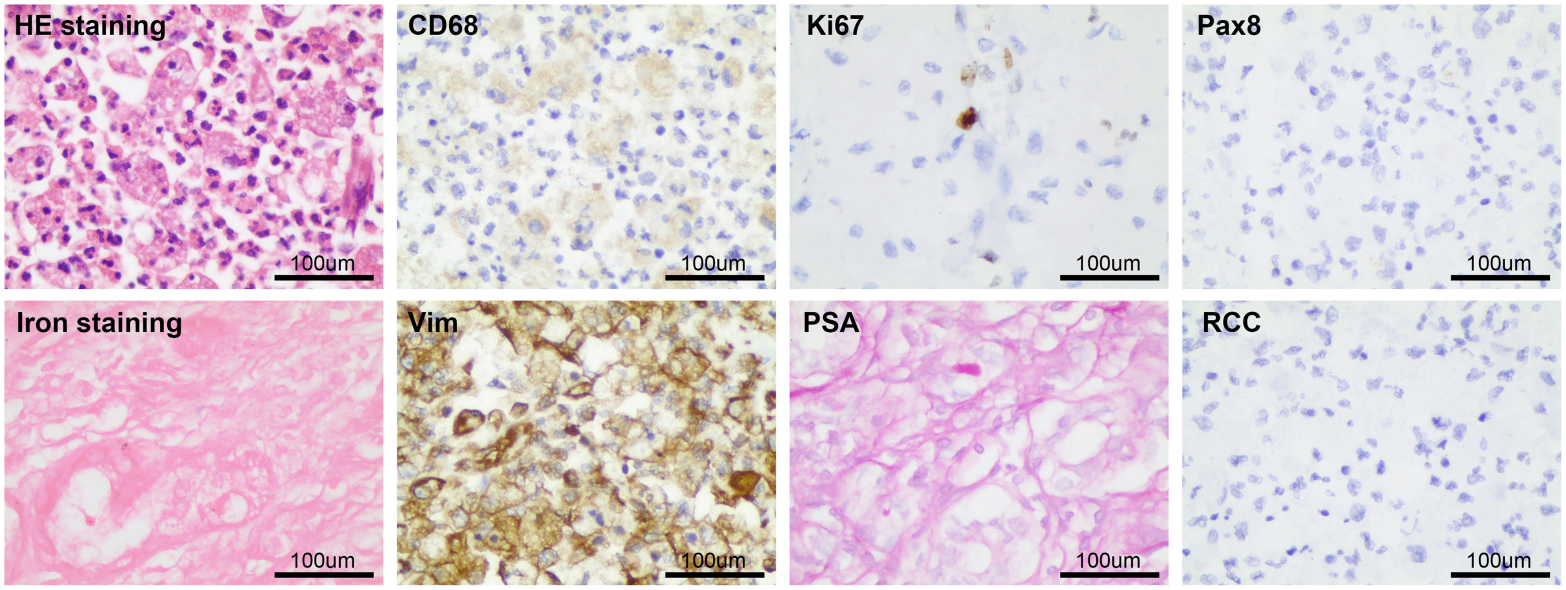
The postoperative pathological examination results were basically consistent with the preoperative puncture pathological examination results.

**Figure 7:**
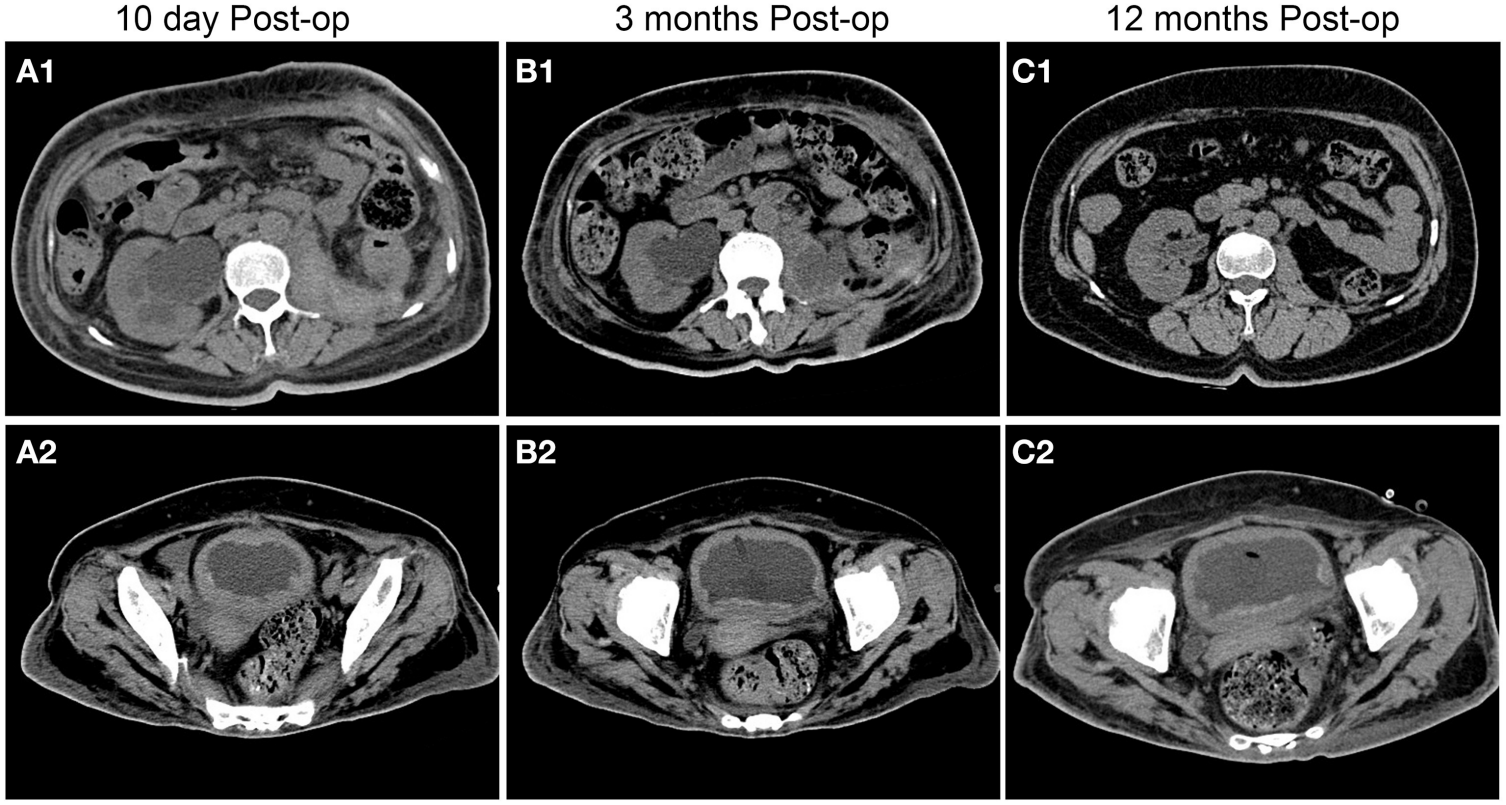
Postoperative follow-up of patients with urinary CT findings. With the extension of time, the right hydronephrosis was gradually relieved, the renal function was gradually improved, and the bladder wall thickening was also significantly improved compared with the preoperative condition.

After anti-infection treatment, the patient's condition did not improve, and fatigue and poor mental condition worsened. Imaging suggested that the bilateral ureters and bilateral hydronephrosis gradually decreased and that the left kidney occupancy persistently increased (Figure 4). After our urological consultation, we considered that the patient's anti-infection and anti-tuberculosis treatments in sufficient amount and course had a poor effect; given the patient's condition, we considered that the left kidney occupancy was probably left renal malakoplakia. After the patient's family was informed of the patient's condition, the patient and her family requested left nephrectomy, and the patient was admitted to the Department of Urology. After the transfer, the laboratory parameters were rechecked, and the anti-infection treatment was continued, along with blood transfusion to correct anemia and nutritional support. The patient's general condition was assessed by the Departments of Anesthesiology, Cardiovascular Medicine, and Respiratory Medicine as tolerable for surgery. Left nephrectomy was then performed under general anesthesia (Figure 5). Right nephrostomy and right ureteral stent placement were not performed because her right hydronephrosis gradually decreased during hospitalization. The postoperative pathological examination of the left kidney showed left renal malakoplakia (Figure 6). The patient recovered well after the surgery; the infection indexes became normal; and the renal function was better than that before. Regular postoperative urinary CT examination showed that with the extension of time, the right hydronephrosis was gradually reduced, the renal function was gradually improved, and the bladder wall thickening was also significantly improved (Figure 7).

After discharge, the patient continued to undergo oral antibiotic treatment and regular outpatient review in our department. She recently underwent review again in our department 1 year after surgery and was in good general condition, with no notable results of laboratory tests, including complete blood cytology and urinalysis. Imaging tests suggested good recovery of the right kidney, right ureter, and bladder with no notable lesions.

## Diagnosis

Malakoplakia is an extremely rare chronic infectious granulomatous lesion, and the first case was reported by Michaelis and Gutmann in 1902 and named by Hansemann a year later (1). It can occur in all organs and tissues of the body, with the urinary system being the most common. Among its subtypes, bladder malakoplakia has the highest incidence (40%). Malakoplakia can also involve the kidney, ureter, prostate, testes, epididymis, and urethra (1–3). Malakoplakia in other systems such as the gastrointestinal tract (12%, second only to the urinary system, with the colorectum being the most common site), female genital tract, lung, skin, central nervous system, adrenal glands, and thyroid gland has also been reported since 1958 (2–6). Middle-aged individuals have the highest risk of malakoplakia. Of them, women are more prone to developing this condition, with a male-to-female incidence ratio of ~1:4. Meanwhile, malakoplakia in children is rare (7).

The pathogenesis of malakoplakia is unclear. It is generally believed to be related to chronic infection and impaired human immune system. Most researchers believe that there are three mechanisms for the pathogenesis of malakoplakia: (1) Impaired function of the human immune system: Malakoplakia mostly occurs in patients who use immunosuppressive drugs after receiving organ transplants, such as the liver and kidney, or in patients with underlying chronic diseases such as acquired immune deficiency syndrome, diabetes mellitus, and tuberculosis (8). In our case herein, the patient had type II diabetes mellitus, did not receive regular treatment, and had poor glycemic control, which was a risk factor for the disease. (2) Infection: The causative organism is mainly *E. coli*, accounting for more than 90% of all bacteria, followed by *Proteus* and *Klebsiella* (1, 3, 9). In our case, the patient had recurrent chronic urinary retention and severe urinary tract infection, and *E. coli* was cultured in urine, blood, and lesion puncture drainage fluid, which is also consistent with the findings of existing studies. (3) Macrophage dysfunction: Macrophages in patients with malakoplakia are unable to digest the germs completely owing to intracellular lysosomal dysfunction, which results in residual undigested bacteria in the macrophages and the accumulation of minerals such as calcium and iron in the bacteria in the macrophages, forming the characteristic lesion Michaelis–Gutmann (M–G) vesicles. This forms the main basis for the diagnosis of malakoplakia (1, 10, 11).

The clinical presentation of malakoplakia varies with the lesion site. Malakoplakia of the bladder is the most common form of urologic malakoplakia and presents mainly with urinary tract irritation and hematuria. Imaging and cystoscopy reveal intravesical occupancies. When the lesion occurs in the upper urinary tract, it may manifest as a mass, swelling and discomfort in the lower back, and fever. When the lesion involves the bilateral kidneys or bilateral ureters and causes obstruction, it can lead to renal insufficiency or even acute renal failure. These urinary symptoms have no specific manifestations and can be easily misdiagnosed as urinary tract infection, tuberculosis, and benign and malignant tumors. No notable symptoms of urinary tract irritation, such as frequent, urgent, and painful urination, were seen during the consultation of this patient; however, there was recurrent gross hematuria. Imaging revealed a thickened and gross bladder wall and a left renal mass. The patient's left kidney and bladder malakoplakia combined with recurrent chronic urinary retention resulted in renal insufficiency, which is consistent with the clinical presentation of urologic malakoplakia.

## Treatments

Malakoplakia is a benign, self-limiting disease that usually has a good prognosis. Owing to its rarity, there are no systemic treatment options for this condition. Based on previous reports of experience in the treatment of this disease, treatment of bladder malakoplakia is currently based on pharmacologic therapy, mainly anti-infection treatment, which can achieve good results. Quinolones are effective against gram-negative bacteria, such as *E. coli*, have high intracellular blood concentrations in macrophages, and are effective in 80–90% of patients with malakoplakia (12). Some studies have also reported that the combination of sulfonamides or rifampicin with quinolone therapy increases the anti-infective effect. Recently, it has also been suggested that cholinergic agents such as methine choline combined with vitamin C can increase the intracellular cGMP/cAMP ratio in macrophages, thereby improving intracellular lysosomal function in macrophages, and it is effective in patients with malakoplakia (13, 14).

Patients with malakoplakia can usually achieve better results with pharmacologic treatment. However, for patients with poor results with pharmacologic treatment, transurethral bladder lesion electrosurgery or electrocoagulation is feasible, and the combination with pharmacologic treatment can achieve better results (15). Upper urinary tract malakoplakia often has a malignant tendency, and once it is diagnosed, aggressive surgical treatment is recommended. Unilateral nephrectomy is feasible in cases of unilateral renal involvement and bilateral nephrectomy in cases of bilateral renal involvement (more than 60% of cases) to preserve renal function (3, 16, 17). Patients with bilateral parenchymal involvement have a poor prognosis and usually die within 6 months (18). In our patient, obstructive renal insufficiency and infection were the main manifestations; bilateral hydronephrosis gradually resolved, and renal function gradually improved after indwelling catheterization. However, the patient's infection was not controlled and progressed rapidly after a full course of full-dose anti-infection treatment, and left renal, left psoas major, and left iliac fossa abscesses formed within a short period. The possibility of urinary tract malakoplakia was considered in relation to the patient's pathological findings of the bladder; thus, left nephrectomy was performed. Considering that the fluid in the right kidney and right ureter gradually decreased, the patient did not undergo right ureteral stenting and right nephrostomy. After the surgery, the anti-infection treatment was continued, and the patient recovered well after 1 year of follow-up.

Ureteral malakoplakia often presents as stenosis, causing obstructive hydronephrosis. It can cause obstructive renal insufficiency if the bilateral ureters are involved (18). Patients with ureteral lesions can be treated medically, and patients with obstruction can be treated with ureteral stenting or nephrostomy to preserve renal function. Excision and re-anastomosis of the ureteral stenosis segment are feasible for single lesions in the middle and lower segments (3).

Malakoplakia of the prostate, testes, and epididymis is rare and difficult to differentiate from other lesions; once it is diagnosed, prostate enucleation or testicular and epididymal resection is recommended (19). Anti-infection treatment with quinolones, sulfonamides, and rifampicin is provided before and after surgery.

## Conclusion

In conclusion, urological malakoplakia, as a rare disease, is clinically difficult to differentiate from infections, tuberculosis, and benign and malignant tumors of the urinary tract. Confirmation of this disease mainly depends on the results of pathological examination. Its diagnosis is difficult, but it should be considered when encountering patients with similar clinical manifestations in clinical practice.

## Data Availability Statement

The raw data supporting the conclusions of this article will be made available by the authors, without undue reservation.

## Ethics Statement

This study has obtained the consent of the patient and his guardian.

## Author Contributions

ZW and JR designed the study, analyzed the data, wrote the manuscript, and reviewed the manuscript. JR collected clinical data. All authors contributed to the article and approved the submitted version.

## Funding

This work was supported by Hainan Department of Science and Technology of China (Grant Number: ZDYF2018104), National Natural Science Foundation of China (Grant Number: 81760138), Ministry of Science and Technology of China (Grant Number: 2013DFA51290), and Hunan Science and Technology Innovation Plan (2018SK2105 and kq1606001).

## Conflict of Interest

The authors declare that the research was conducted in the absence of any commercial or financial relationships that could be construed as a potential conflict of interest.

## Publisher's Note

All claims expressed in this article are solely those of the authors and do not necessarily represent those of their affiliated organizations, or those of the publisher, the editors and the reviewers. Any product that may be evaluated in this article, or claim that may be made by its manufacturer, is not guaranteed or endorsed by the publisher.
